# Leading from behind the scenes: Community birth attendants, the unsung heroines of childhood vaccination in Ibadan urban slum communities

**DOI:** 10.1371/journal.pgph.0006691

**Published:** 2026-06-16

**Authors:** Folusho Mubowale Balogun, Olubukola Christianah Omobowale

**Affiliations:** 1 Institute of Child Health, College of Medicine, University of Ibadan, Ibadan, Nigeria; 2 University College Hospital, Ibadan, Nigeria; 3 Department of Community Medicine, Faculty of Clinical Sciences, University of Ibadan, Ibadan, Nigeria; Johns Hopkins University Bloomberg School of Public Health, UNITED STATES OF AMERICA

## Abstract

Community birth attendants (CBAs) represent an insufficiently integrated resource in childhood immunization programs, particularly in urban slum communities where healthcare access remains challenging. Their role as birth attendants has been well documented, however, understanding their role in infant vaccination uptake is crucial for improving immunization uptake and child welfare. This study explored the knowledge of CBAs about infant immunization and assessed their roles in promoting vaccination uptake in urban slum communities of Ibadan, Nigeria. A narrative study design was employed across eight urban slum communities in three local government areas of Ibadan. Six focus group discussions were conducted with 37 CBAs using an interview guide developed in Yoruba language. Data were analyzed using content analysis, guided by the knowledge, attitude and practice model. CBAs had a good understanding of immunization as a disease prevention strategy and had comprehensive knowledge of birth dose vaccines’ (BCG, OPV, Hepatitis B) names and timing. However, knowledge about the other immunization visits’ timing varied considerably among them. CBAs generally had positive attitude towards immunization. They played diverse roles in immunization process including community mobilization for immunization campaigns, health education, referral to immunization clinics, appointment reminders, identification and support of zero-dose children, and providing transportation assistance when mothers were unable to attend clinics. Their trusted position within communities and informal collaboration with primary health centers positioned them as vital intermediaries in vaccination uptake. Key barriers included limited vaccine knowledge, lack of formal recognition by healthcare workers, and poor self-efficacy in navigating vaccination procedures. Despite knowledge gaps and systemic barriers, CBAs serve as crucial facilitators of infant immunization in urban slum communities in Ibadan, through their trusted relationships and comprehensive support activities. Formal integration of CBAs into immunization programs, coupled with targeted training and recognition, could significantly enhance vaccination coverage in disadvantaged urban populations.

## Introduction

The child health indices in many urban slum communities in developing countries are suboptimal and many times worse than that of the other urban and rural children [1,2]. About a third of African urban population live in urban slums [3] and the health of the children in this population deserves great attention. Vaccine preventable diseases rank high among the leading causes of under-five mortality in urbans slums but infant vaccine uptake in the slum communities have been shown to be abysmally low [4]. In Nigeria, infant immunization coverage remains suboptimal despite numerous past and ongoing interventions to address the problem. The Nigerian Demographic Health Survey (NDHS) of 2023/2024 reported that immunization coverage among children aged 12–23 months improved from 31% in 2018 to 39% in 2023/2024, while zero dose children reduced minimally from 36% to 31% [5]. Different studies have shown that mothers with no formal education and who are from the least wealth quintile strongly predict incomplete or no immunization among Nigerian children [1,6]. These characteristics are prevalent in urban slums which are also plagued with other factors that increase under-five mortality, including inadequate housing, poor sanitation and overcrowding.

There have been efforts to improve the overall immunization in Nigeria with a lot of investments in the supply and demand end. Also, partners like GAVI and UNICEF have been engaged to ensure steady supply of vaccines [7]. The cold chain system of the different states of the country has been optimized and they receive active support from the government for optimal functioning [8]. In addition, coordinated community involvement and sensitization is being done regularly to drive the demand for vaccines and active steps are taken to protect vaccinators from harassment and violence in conflict prone areas [9,10]. Active data collection is also deployed to monitor the progress of infant immunization. Despite all these steps, immunization in urban slum communities remains suboptimal. This calls for more effective strategies that could improve urban slum immunization uptake because, no group of people should be left behind based on the objectives of the sustainable development goals.

In this study, the term “Community Birth Attendants” (CBAs) is used to describe indigenous middle age/older women who provide traditional antenatal, intrapartum, and postnatal care within their communities, operating outside the formal health system [11]. While the term “Traditional Birth Attendants” (TBAs) is more widely used in the Nigerian health system lexicon, we adopt the term CBA as it is consistent with usage in the Nigerian peer-reviewed literature [12] and with the broader parent study from which this dataset is drawn. The CBAs in this study are equivalent to TBAs as conventionally described: their practice is guided by local customs, personal experience, and apprenticeship, but unlike TBAs, they are not licensed birth attendants. They also live in the communities where they practice just like Traditional birth attendants, and they provide child health services too, especially in remote and underserved communities [13]. CBAs operate usually independent of the formal health system [14]. However, the populace sometimes prefers their services even when they have access to formal health care facilities. The most recent NDHS showed that Nigerian women aged 15–49 years who had antenatal care from skilled health providers decreased from 67% in 2018 to 63% in 2023/2024 [5]. Also, only 43% of live births took place in health facilities, and another 43% had postnatal checks in the first two days after birth. There is a high chance that the women who did not receive the required care before, during and after delivery from health facilities patronized CBAs. Different reasons given for the preference of CBA services include trust, easy access and inexpensive services [14]. They have been identified as a vital connection to the achievement of universal health coverage in Africa [15] with potential to improving maternal health indices [16]. Based on their influence, CBAs may be able to contribute positively to the uptake of infant vaccination in urban slums. There was a report of reduction in neonatal mortality in Zambia following the training of CBAs in neonatal resuscitation, the use of single dose amoxicillin for infection prophylaxis and referral of newborn who need further care to health facilities [17]. An earlier Nigerian study also demonstrated the willingness of CBAs to promote vaccine uptake following training to improve their knowledge about vaccines [18].

The primary health centers where immunization of most children in Nigeria take place have a lot of shortcomings that negatively affect immunization uptake. Most PHC are understaffed [19] despite the busy clinic schedule that they operate. This results in little or no time for community outreaches to drive the demand for immunization. Major gaps have been identified in earlier interventions to improve immunization which raises the question of how to deliver effective and sustainable routine vaccine uptake in Nigeria [20]. Oyo state has a large proportion of her population in urban slums and the state has the least proportion of children who had received the basic infant vaccines and the highest number of zero dose children in South West Nigeria [5]. A multi component intervention may be able to improve the immunization of children in Oyo state but this will require some baseline data to guide the design. This study therefore explored the knowledge and attitude of the CBAs in Ibadan, the capital of Oyo state, about infant immunization and described their roles in infant immunization programs in selected slum communities. This is a part of a larger study that sought to design an intervention to improve infant immunization timeliness and completion in urban slums through the involvement of older women in immunization process [21,22]. This study contributed to the knowledge base regarding the existing structures that support infant immunization in the urban slum communities, and the findings were used to design the intervention to improve infant immunization uptake in the slum communities.

## Methods

### Ethics statement

The study proposal was approved by the Oyo State Research Ethics Review Committee (AD 13/479/784). Written informed consent was obtained from each participant, and no identifier was used during data collection and analysis. In addition, ethics approval required community-level anonymity to protect the identities of participants and the communities in which they reside, given the small and identifiable nature of these communities. Accordingly, the specific names of the study communities have been withheld throughout this manuscript.

### Study area

This study was conducted in eight urban slum communities located in three (out of the five) urban local government areas (LGAs) of Ibadan, the capital of Oyo State, southwest Nigeria. These LGAs are Ibadan North, Ibadan North East and Ibadan South East local government areas. Each LGA is divided into wards for administrative purposes resulting in 12 wards in each LGA. Also, each ward has one primary health center (PHC). In addition, there are some private health facilities in these LGAs, however, the actual number of private facilities is difficult to ascertain as the registries with the LGA secretariats are usually not up to date. Some of the private health facilities might have closed down or new ones might have gotten established without registering with the LGAs. There is also one secondary facility owned by the Oyo State government and one tertiary health care facility owned by the federal government in Ibadan North LGA. Both facilities are accessible to residents in the other LGAs. The communities qualify as urban slums based on the UN-Habitat criteria, which defined an urban slum as characterized by high population density, inadequate sanitation infrastructure, predominantly informal and substandard housing, limited access to basic amenities, and documented suboptimal health outcomes [23]. Specifically, low immunization coverage in these communities has been reported in our previously published work [4], and comparable communities within these LGAs have been formally characterized as urban slums in the published literature [2].

### Study design and study population

Narrative study design was used in this study. The study population were the CBAs who traditionally provide antenatal and postnatal services for pregnant women in the communities. The CBAs live in the communities and are usually well known to community members. They have informal affiliations with primary health centers around them where they send pregnant women who require medical interventions during pregnancies and deliveries.

### Data collection instrument

Data was collected using an interview guide developed by the researchers, based on their knowledge and experiences. The interview guide was in Yoruba language, the indigenous language spoken in the research area. Content validity was assessed by the subject matter experts to confirm that the guide adequately covered the domains of interest. Also, face validity was assessed by subject matter experts in public health and immunization, as well as CBAs from a LGA different from the three study LGAs, to ensure the questions were clear, culturally appropriate, and comprehensible to the target population.

### Data collection procedures

The data collection started on July 6, 2018, and ended on July 12, 2018. All the existing CBAs were identified through a community scout. There were 37 CBAs in all, and they all agreed to participate in the study. They were then divided into six groups (six per group and a group had seven participants) for the focus group discussion (FGD). The FGDs were conducted in a hall located in one of the study communities. Three research assistants (two females and one male) who had no prior interactions with the CBAs were involved with the data collection (functioning as a moderator, a timekeeper and a notetaker). The research assistants all had masters degree in Public Health, and were full time research staff at the time of data collection. The research assistants had vast experience in conducting qualitative research interviews and they had a refresher course in research ethics and qualitative data collection before the commencement of data collection. Also, both authors are medical doctors (FMB is a pediatrician and OCO is a community physician) and both had no contact with the CBAs before and all through the data collection period to avoid biases: confirmation bias on the part of the authors and both social desirability and sponsor biases of the CBAs. Each participant provided written informed consent after the study was introduced to them and their demographics were taken. All the FGDs were conducted in Yoruba language and recorded with two digital audio recorders. Both authors and all three research assistants are native Yoruba speakers, ensuring linguistic fidelity throughout data collection. The notetaker documented the demographic characteristics of the participants, the nonverbal cues and the details of the group dynamics. Probes were used to get clarifications as necessary. Member checking was done at the end of each interview to ascertain the correctness of their responses. Each FGD lasted between 38–60 minutes. There were no repeat interviews, and non-participants were not present during the FGDs.

### Knowledge, Attitude and Practice (KAP) model

The Knowledge, Attitude and Practice (KAP) model was proposed by Mayo in the 1950s [24]. The KAP model posits that knowledge (of a phenomenon or object) and the attitude determine the behavior (decision making) of individuals. The relationship between the three components may not always be linear as they can be influenced by external factors like the context and other individual characteristics [25]. The KAP model is commonly used to assess behavioral determinants which influence the use of preventive and promotive health services. It is useful in identifying knowledge gaps, misconceptions, group perceptions and typical behaviors toward specific health services, and these may promote or hinder the uptake of such services by specific populations [25]. Therefore, KAP have been identified as critical components of behavioral change models [26]. Using this model for the data analysis can highlight critical areas which can be targeted to support the activities of the CBAs in promoting the uptake of infant immunization.

### Definition of terms

Knowledge: For this study, knowledge refers to what the CBAs know about infant immunization, and this may be correct or incorrect knowledge.

Attitude: This refers to the feelings that the CBAs have towards infant immunization, and it may be positive or negative attitude.

Practice: This includes all the steps and activities that the CBAs engage in to promote or hinder infant immunization uptake.

### Data analysis

Data was analyzed using content analysis using the Knowledge, Attitude and Practice (KAP) model.

All FGD recordings were transcribed verbatim in Yoruba and subsequently translated into English. A back-translation process was employed to minimize translation errors and information bias: the translated transcripts were independently back-translated into Yoruba by a team member who was not involved in the original translation, and discrepancies were resolved through discussion. Each transcript was read critically by two research assistants and one of the researchers (OCO) and codes were generated inductively and deductively using the KAP model. These codes were reconciled by the three coders in a meeting where discrepancies were discussed and resolved. Thereafter, codes with similar concepts were grouped together to form subthemes and themes based on the components of the KAP model. Iterative comparison was made within each theme to ensure that each can stand as a theme. There were also comparisons between themes to unveil the patterns among them. Quotes that represented each identified theme were then selected from the transcripts.

This manuscript was prepared using the Consolidated criteria for Reporting Qualitative research (COREQ) checklist (S1 Checklist).

### Underlying data

The data associated with this manuscript can be found at in Mendeley Data, V1, https://data.mendeley.com/datasets/hyp5npbw9y/1 [22].

## Results

There were 37 CBAs who participated in six FGDs, all were females and married. Their age ranged from 20 to 70 years, and no variation was observed in their responses based on their age. The following were the identified themes from the FGDs:

- CBA’s relationship with community members
- Knowledge about infant immunization
- Attitude towards infant immunization
- CBA’s practices that support infant immunization

### CBA’s relationship with community members

The CBAs had close relationships with the community members because they lived within the community, and they were familiar with the people. The community members also had easy access to them and trust their judgements, making them the first port of call when seeking solutions to their health problems. These are illustrated in the quotes below.

Most people, if they have any health issues, they do not want to go to the health center, they will first come to our facility. FGD 06, p01

If anything happens to them, they don’t usually take them to the hospital, they will first bring them to us and wait for what we would say. They believe in us and what we tell them to do; we can even follow them to the hospital to see a doctor. FGD 03, p05

The CBA also had some informal collaboration with the PHCs based on the recognition of their influence on community members by the PHC workers. The following quotes support this.

The health workers even use to tell us that once our registered pregnant women deliver their babies, we should quickly refer them to the health center for immunization. FGD 05, p03

They know that many people do visit the health center based on our recommendation. FGD 05, p01

We can even call the doctors to notify them that a child is coming for consultation and treatment. FGD 03, p05.

### Knowledge about infant immunization

All the CBA correctly stated that infant immunization is meant to prevent childhood diseases as seen in the quotes below.

It prevents some diseases. It’s like a soldier fighting against diseases. FGD 01, p03

It prevents all diseases that affect children like polio, tuberculosis, whooping cough, the diseases that deform children. FGD 04, p06

The knowledge about the timing of visits for infant vaccination was highly variable within and between the groups, but few CBAs stated the timing of the immunization visits correctly as seen in the quote below.

They will receive BCG, OPV and Hepatitis B at birth, and another set at 6 weeks, then 10 weeks, another one at 14 weeks and later measles at 9 months. FGD 06, p03

However, all the CBA were familiar with the birth dose vaccines as shown below.

When a baby has just been delivered, we would take the baby to the health center to receive the BCG, OPV and hepatitis vaccine… FGD 05, p01

### Attitude towards infant immunization

Generally, the CBA had a positive attitude towards infant immunization. And this is seen in their various positive inclinations towards both the infant vaccines and the immunization program. They had positive belief in what immunization can do to improve under five mortality as seen in the quotes below.

The program is very good… if the program continues, it will reduce the infant’s mortality’ FGD 03, p2

It is important for mothers of infants to immunize their children as recommended. FGD 05, p2

They prioritized infant immunization and this was the attitude behind the extra efforts that they put in to ensure that infants in their communities are immunized as seen in the quote below.

We monitor mothers by following them to the health center to get their infant immunized. FGD 02, p4

The CBAs also liked the health care staff in charge of immunization and they were happy with the way they and their clients were being treated in immunization clinics. This was illustrated in these quotes:

…they treat us well, when we take newborn to them, they don’t delay us. FGD 01, p6

They attend to us irrespective of whether our pregnant women deliver in their health center or not. FGD 06, p3

Lastly, the CBAs were willing to support immunization programs to ensure optimal infant immunization in their communities. This was shown in the following quotes:

The government should also employ the community birth attendants for immunization mobilization because we know the people… we can easily convince them to take the immunization. FGD 01, p6

…we are ready to cooperate with them in our local government. FGD 06, p3

### CBA’s practices that support infant immunization

The CBAs had many roles that they play in infant immunization as shown by the subthemes in Table 1. Some of them had been employed as ad hoc staff during immunization campaigns where they mobilized community members for immunization. They were also invited for community mobilization based on the recognition of their influence on community members. They give health education about immunization during community meetings and in churches and mosques. They refer new mothers to

**Table 1:** Subthemes showing the different roles that CBAs play in infant immunization in their communities.

Subtheme	Relevant quotes
Mobilization for immunization campaigns	I was involved in the mobile immunization services in the communities, I would go early to inform mothers in the communities that the immunization people are coming so that mothers will bring out their children for immunization FGD 04, p01
	The health workers come to our area frequently…we do mobilize people to take the immunization. FGD 01, p07
Referral to immunization clinic	We advise them to immunize their children, and some people listen once we talk to them… FGD 05, p05
	I also direct the mothers to the different immunization centers so they can take their children to for immunization. FGD 04, p04
Immunization health education	We would educate them on the importance of immunization…we would ask them to inform new nursing mothers at home to immunize their children… at times, we would go to mosques and churches to talk about the need to take vaccines. FGD 03, p05
	Pregnant women that deliver their babies at our place, we do tell them to immunize their children and warn of the dangers of not immunizing their children FGD 04, p04
Reminders about immunization visits	We have their date of births on record in the office, and this makes it easy for us to follow them up and ask them if they have immunized their children or not… some of them live in the same vicinity with us and this makes it easy and convenient for us to see them. If we don’t monitor them, they wouldn’t take it. FGD 03, p05
	I normally request for their immunization card to know if they are telling the truth. FGD 03, p04
	We can go there about 3 times to remind her to immunize her baby. I do that in my community with new mothers. We can do that as an act of kindness to other people. FGD 04, p01
Identify zero dose children	There was a woman who had 4 children; we discovered that the first 3 were never immunized. We took them to the health center, and they immunized them though they couldn’t take the birth dose again… FGD 01, p06
Other personal support for immunization	…even when the mother is weak, we carry the baby, get them immunized and get cards for them so that they can continue when they get home. FGD 01, p06
	…we do go to their houses once they could not be reached on phone to remind them of their child’s immunization. FGD 01, p04

immunization clinics following the delivery of babies and remind them to complete the schedule during home visits and through phone calls. They also assist in taking infants of sick mothers to the clinics for immunization. In addition, they identify zero dose children in their communities and assist them to get immunized.

## Discussion

This study set out to explore the knowledge and attitude of CBAs about infant immunization and assess their roles in promoting vaccination uptake in urban slum settings in Ibadan, Oyo state. Key findings reveal that CBAs in urban slum communities of Ibadan function as vital, yet largely unrecognized, facilitators of infant immunization. Despite operating outside formal healthcare structures, CBAs show appreciable knowledge about vaccination benefits and engage in diverse activities that directly support immunization uptake in their communities.

We found out that all CBAs correctly understood immunization as a disease prevention strategy and this aligns with previous research among traditional birth attendants in Kenya, where TBAs provided advice on immunization alongside other maternal and child health services [27]. Similarly, studies from post-conflict settings in Burundi and northern Uganda demonstrated that TBAs serve as the first point of contact for pregnant women when formal healthcare systems are compromised [28]. The comprehensive knowledge of the names and timing of the birth dose vaccines shown the CBAs in this study is particularly significant, as Hepatitis B vaccine require immediate administration following delivery to effectively prevent mother to child transmission of Hepatitis B virus, an infection that is endemic in Nigeria. The birth time is a critical window when CBAs are most actively involved in newborn care in underserved areas in Nigeria because they are closer to the grassroots and they are usually the first port of call [29,30]. There was a high variability of the knowledge about the other immunization visits’ timing amongst the CBAs which could lead to passing of wrong immunization timing to their clients, resulting in missed or incomplete infant immunization. However, findings from an earlier systematic review showed that structured training programs for community health workers significantly improved their knowledge and performance in vaccination programs [31]. This suggests that targeted educational interventions could enhance CBAs’ effectiveness as vaccination advocates with regards to correct messaging for timing of immunization visits.

The general positive attitude of the CBAs towards infant immunization could be leveraged to improve infant immunization in the slum communities. This is because attitude is an important precursor of performing a behavior [32] and the positive attitude of the CBAs in this study provides an opportunity to equip them with the right immunization training which can improve their knowledge of immunization, transforming them to strong and competent agents for the promotion of infant immunization in their communities. Negative attitude of health care workers has been linked to their poor uptake of immunization [33,34] and this could impact on their messages to their clients because health workers are trusted source of immunization messages by community members. There have been reports of healthcare workers withholding immunization messages from families or discouraging uptake of vaccines due to their negative attitude towards vaccines [35,36].

The roles performed by CBAs ranged from community mobilization to zero-dose child identification which aligns with a recent systematic review demonstrating that community health workers who are trusted by under-reached communities can increase equity in vaccine access for under-immunized, and zero-dose children [31]. This finding is particularly relevant given that suboptimal infant vaccination is common in Nigerian urban slums, with vaccination indicators reported to be worse in these settings compared to other urban areas [4]. Our study’s finding of CBAs’ role in zero-dose child identification represents a particularly valuable contribution that can address urban slum vaccination challenges. A systematic review and meta-analysis on community engagement in vaccination promotion found that community-based interventions could significantly improve vaccination coverage, particularly when delivered by trusted community members [37]. This supports our finding that CBAs in urban slums in Ibadan, who are highly trusted within their communities, could serve as effective vaccination promoters.

Similarly, the appointment reminder services provided by CBAs in our study echo findings from a meta-analysis of interventions to improve immunization coverage among children and adolescents, which demonstrated that reminder systems can increase vaccination coverage by significant margins [38]. However, our study reveals that these reminder services are provided informally by CBAs, suggesting potential for systematization and integration into formal healthcare systems. The CBA-community relationships highlight an advantage in vaccination promotion that aligns with broader literature on community health interventions. The community members’ preference for consulting CBAs before seeking formal healthcare agrees with findings from research in conflict-affected settings where TBAs became primary healthcare providers due to their trusted status and accessibility [27]. This trust-based referral system represents what Amref Health Africa describes as the traditional role of TBAs who are highly respected healthcare providers in African communities [38]. However, our findings show how trust translates into specific vaccination-related activities in urban contexts. The informal collaboration between CBAs and primary health centers, while valuable, remains ad hoc and unsystematically formalized compared to more structured community health worker programs seen elsewhere. Research from UNICEF’s work in Nigerian urban slums confirms the challenges of reaching zero-dose children and highlights the importance of community-based approaches to overcome barriers that prevent mothers from vaccinating their children [39]. Our study provides specific evidence of how CBAs address these barriers through their trusted community positions.

Some caution must be applied if the integration of CBAs as advocates and support for immunization programs are to be considered. This is because earlier studies have reported the overstep of boundaries in CBA’s operations outside the provision of care for pregnancy and delivery [27]. This can result when they do not have adequate knowledge of immunization as seen in this study and when their activites are not closely monitored. If effective structures can be instituted to monitor and regulate the practices of CBA, their relationship with the community members can become an invaluable resource that can be used to promote infant immunization uptake. The downside of the CBAs involvement in infant vaccination including limited knowledge of vaccine timing may appear as a limitation in their activities and usefulness. However, a systematic review examining the cost-effectiveness of community health workers for childhood vaccination in similar settings found that these workers can be effective, and their impact can be significantly enhanced when they receive appropriate training [40]. The knowledge gaps identified among CBAs, particularly regarding vaccine timing and specific vaccine functions, contrast with findings from research on formal community health worker programs where structured training protocols led to improved performance [30]. These gaps indicate that any prior general community health training received by CBAs has not fully translated into immunization-specific competencies, supporting the need for targeted refresher interventions focused on vaccination schedules and immunization program protocols. The lack of formal recognition of CBAs specifically within immunization program structures represents a missed opportunity, a gap that has been identified in other African contexts [13,16].

Research on TBAs in post-conflict settings showed that when they received some form of training, their effectiveness in maternal and child health services improved significantly [27]. Meta-analytic evidence suggests that behavioral interventions, particularly provider recommendations and community-based approaches, can significantly increase vaccine uptake [41]. Our findings indicate that CBAs already engage in many of these evidence-based strategies informally, suggesting that formal integration and regulation could amplify their impact. The World Health Organization’s emphasis on community engagement in vaccination programs aligns with our findings showing CBAs’ effectiveness in community mobilization and health education [31]. However, research indicate that the success of community health worker interventions depends heavily on appropriate training, supervision, and integration with formal health systems, and these are currently lacking in these CBAs’ vaccination activities [30]. Therefore, tested structures are required to meet the knowledge deficiencies of the CBAs regarding vaccines, and their activities should be closely supervised and regulated for effective impact on infant immunization coverage if they are to be integrated into immunization programs.

### Study limitations and future directions

This study’s focus on urban slum communities in a single Nigerian city limits generalizability to other settings, though findings align with research from similar contexts across sub-Saharan Africa. The exclusively qualitative approach, while appropriate for exploring roles and relationships, could not quantify the impact of CBAs’ activities on vaccination coverage, unlike systematic reviews that provide measurable effect sizes for community health worker interventions [26]. Also, the use of FGD for data collection might have influenced the responses of some CBAs who could have given socially desirable responses. This may be responsible for the overwhelming positive attitude towards infant immunization. Future research should examine vaccination outcomes in communities with active CBA involvement compared to those without, explore optimal training and integration models based on successful community health worker programs, and investigate the cost-effectiveness of formal CBA integration compared to other community-based vaccination strategies documented in the literature. Additionally, while this study’s objectives were specifically to explore CBAs’ knowledge and roles in infant immunization and the FGD guide was designed for that, future research should investigate the reasons underlying community preference for CBA services specifically in the context of immunization. Understanding whether the trust and accessibility that drive preference for CBAs in general maternal care extends to immunization uptake decisions would provide important evidence for designing CBA-integrated immunization interventions. Lastly, the time difference between the collection of data and the writing of this manuscript could have reduced the relevance of the findings in this study. However, infant immunization coverage is still suboptimal in Ibadan [42] with persistent infrastructural deficits and manpower shortages [43]. Also, there is no known novel strategy that had been introduced to improve infant immunization uptake in these slum communities since the data collection and the present.

## Conclusion

Community birth attendants represent an insufficiently integrated resource for improving infant immunization coverage in urban slum communities of Ibadan. Despite operating with limited formal recognition and facing systemic barriers, they demonstrated substantial commitment to vaccination promotion and possess unique community access that formal healthcare systems often lack. The evidence from this study suggests that strategic integration of CBAs into immunization programs, supported by immunization-specific training, supervision and regulation, in addition to formal recognition within vaccination program structures, could significantly enhance vaccination coverage and equity in disadvantaged urban slum populations. The trusted relationships and comprehensive support activities demonstrated by CBAs position them as crucial bridges between communities and formal healthcare systems in achieving optimal immunization outcomes.

## Supporting information

S1 Checklist COREQ 32 – Item checklist. (DOCX)
